# Evaluating pneumoperitoneum pressure in robotic liver surgery: a propensity-score matched analysis in a high-volume center in Scandinavia

**DOI:** 10.1007/s00464-025-12283-2

**Published:** 2025-10-17

**Authors:** Daisuke Fukumori, Christoph Tschuor, Takashi Hamada, Paul Suno Krohn, Stefan Burgdorf, Øivind Jans, Hans-Jørgen Frederiksen, Jens Hillingsø, Peter Nørgaard Larsen

**Affiliations:** 1 Department of Surgery and Transplantation, Rigshospitalet, Copenhagen University Hospital, Copenhagen, Blegdamsvej 9, 2100 Copenhagen, Denmark; 2 Department of Anaesthesiology, Center for Cancer and Organ Diseases, Rigshospitalet, Copenhagen University Hospital, Copenhagen, Denmark

**Keywords:** Robotic liver surgery, Pneumoperitoneal pressure, Liver resection, Laparoscopic liver surgery

## Abstract

**Background:**

Minimally invasive liver surgery usually involves the use of standard pneumoperitoneal pressure (12–15 mmHg) and low central venous pressure (CVP) management to reduce intraoperative bleeding while hereby possibly increasing the risk for gas embolism. The purpose of this study is to evaluate the efficacy and safety of low pneumoperitoneum pressure (Low-PP: 10 mmHg) compared to standard pneumoperitoneum pressure (Standard-PP: 12 mmHg) in patients undergoing robotic liver surgery (RLS) without active CVP management.

**Methods:**

A single-center retrospective cohort study was conducted from June 2019 to February 2024. Propensity-score matching analysis (1:1) was performed based on age, sex, BMI, ASA classification, diagnosis, and extent of resection (minor or major) for Low-PP group to Standard-PP group. The primary outcome were estimated blood loss (EBL), operating time (OT), length of stay (LOS), and complications (Clavien–Dindo classification).

**Results:**

Before the propensity-score matching (PSM) analysis, the Low-PP group comprised 63 patients and the Standard-PP group comprised 130 patients. Following PSM analysis, each group comprised 62 patients. The pringle maneuver was performed significantly more frequently in the Low-PP group (87.1% vs 50.0%, *p <* 0.001). There were no statistically significant differences with regards EBL, OT, LOS, or overall/major complications between the two groups. Intraoperative anesthetic parameters were comparable, and no signs of gas embolism were observed in either group. In a subgroup analyis for minor and major resections, no statistically significant differences were observed in perioperative outcomes between the groups.

**Conclusion:**

Our study did not find any statistically significant difference in perioperative outcomes of patients undergoing RLS at a pneumoperitoneal pressure of 10 mmHg versus 12 mmHg. We therefore conclude that performing RLS using a pneumoperitoneal pressure of 10 mmHg PP is feasible and safe. Randomized controlled trials are needed to further investigate the potential and benefit of this strategy.

**Supplementary Information:**

The online version contains supplementary material available at 10.1007/s00464-025-12283-2.

Minimally invasive liver surgery (MILS) has become increasingly widely adopted over the past 30 years due to its advantages, such as reduced blood loss, fewer complications, and shorter hospital stays [1–3].

Robotic liver surgery (RLS) was first introduced by Giulianotti et al. [4] in 2003 and considering the body of evidence from various reports of RLS studies to date, a systematic review and meta- analysis of RLS and conventional LLS has since concluded that the techniques are equivalent [5]. Many studies report that a consensus is developing on the safety of RLS, and surgeons increasingly choose the robotic approach for liver resection [6, 7]. Although RLS has demonstrated safety and efficacy equivalent to laparoscopic liver surgery (LLS) [5], intraoperative bleeding during liver parenchymal dissection remains a significant concern in MILS [8, 9].

Low central venous pressure (CVP) management and maintenance of stable intra-abdominal pressure (IAP) are currently recommended as strategies for bleeding management in MILS [10]. However, recent randomized data suggest that the impact of low CVP management during liver parenchymal dissection may have been overestimated [11]. In fact, most centers maintain pneumoperitoneum pressure (PP) at 12 to 15 mmHg and perform liver parenchymal dissection under low CVP management during MILS [12], however this management may increase the PP-CVP gradient and increase the risk of gas embolism [13].

Recent studies have recommended setting PP between 6 and 10 mmHg in general laparoscopic surgery [14, 15]. In addition, low-PP may reduce postoperative pain and prevent liver and kidney injury [16]. However, there is currently no consensus on the optimal PP and CVP management in MILS.

The aim of this study was to evaluate the effects of reducing the PP to 10 mmHg on anesthetic parameters, intraoperative blood loss, and short-term postoperative outcomes in RLS without active CVP management.

## Materials and methods

Indications for liver resection were determined at multidisciplinary team (MDT) meetings (hepatobiliary and pancreatic surgeons, radiologists, medical oncologists) in accordance with Danish Cancer Plan II. Standard work-up included contrast-enhanced CT or MRI of the abdomen and thorax within 4–6 weeks before surgery to assess tumor characteristics and future liver remnant. RLS was introduced stepwise following the 2014 Morioka consensus for LLS and the IWATE difficulty score: initially minor resections in anterolateral segments for benign or small malignant lesions without major vascular involvement, then gradual expansion to posterosuperior segment resections, major hepatectomies, and selected cases after limited prior abdominal surgery as experience increased [7, 17]. During the study period (June 2019–February 2024), RLS accounted for approximately 10–15% of all liver resections on average [7, 17].

**Fig. 1:**
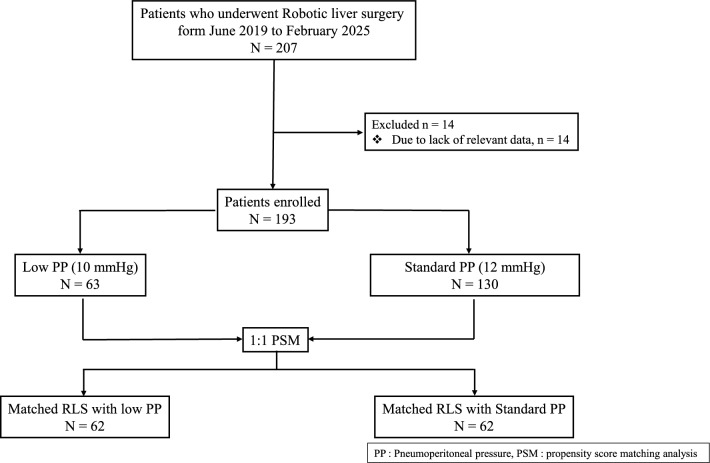
The flowchart shows how the study patients were selected between June 2019 and February 2024, a total of 207 patients were selected

We retrospectively evaluated patients who underwent RLS between June 2019 and February 2024. The number of cases included is described in the results section. RLS was performed using the da Vinci Surgical System® (Intuitive Surgical, San Jose, California, USA) while maintaining IAP by the AirSeal® system. Of the 193 patients, 63 underwent RLS using a Low-PP of 10 mmHg while 130 underwent RLS with a Standard-PP of 12 mmHg. The short-term surgical outcomes of the two groups were compared using a 1:1 propensity-score matching (PSM) case–control design (Fig. 1). PSM used age, sex, BMI, ASA score, diagnosis and extent of resection (minor or major) as matching variables. In addition, subgroup analyses were also performed for patients who underwent minor liver resection, major liver resection, and for patients with a BMI > 30 kg/m^2^.

Patient selection criteria for RLS included liver function, tumor characteristics, and individual patient characteristics. Patients eligible for liver resection at our centers must have a Child–Pugh score of A to ensure adequate liver function. At the time of induction for RLS, patients should have a tumor diameter of less than 5 cm, sufficient remnant liver volume, no portal hypertension, no anatomical variations. With increased experience, indications for liver resection have been extended to patients with larger tumor diameters, multiple tumors and anatomical variations [7, 17].

### Pre-operative assessment and post-operative surveillance

Indications for surgery included malignant and benign liver tumors. Malignant tumors included hepatocellular carcinoma, intrahepatic cholangiocarcinoma, gallbladder cancer, colorectal liver metastases and non-colorectal liver metastases. Benign tumors included hemangiomas, adenomas, and focal nodular hyperplasia. CVP, end-expiratory partial pressure of carbon dioxide, positive end-expiratory pressure (PEEP), peak airway pressure, and estimated blood loss (EBL) was obtained from anesthetic records. Operative time (OT) was defined as the time from skin incision to wound closure. No adjustment was made for adhesiolysis; postoperative complications occurring within 60 days were classified according to the Clavien-Dindo classification. Major complications were defined as events requiring surgical, endoscopic or radiological intervention (Clavien–Dindo classification grade ≥ III).

### Anesthetic management

General anesthesia was administered according to a standardized institutional protocol. Anesthesia was typically induced and maintained with propofol, remifentanil, and neuromuscular blockade. In selected cases with limited liver reserve or cirrhosis, sevoflurane was used as an alternative. All patients received both arterial and central venous catheters for continuous hemodynamic monitoring. Lung-protective ventilation strategies were used, with PEEP set at 5 cm H₂O and tidal volume at 8 ml/kg. The protocol included intermittent reduction of intra-abdominal pressure and pneumatic compression to minimize adverse effects on visceral circulation and preserve pulmonary and renal function.

### Definitions

This study adopted internationally accepted definitions for pneumoperitoneum (PP) settings, surgical complexity, and gas embolism parameters. According to international guidelines or based on previous studies define low-PP as 6–10 mmHg, while conventional PP in laparoscopic liver surgery (LLS) is 12–15 mmHg [18–22]. According to these criteria, low-PP was defined as 10 mmHg and standard-PP as 12 mmHg in this study. Surgical difficulty was assessed using the IWATE criteria proposed by Wakabayashi et al. [23]. Minor liver resection was defined as resection of two or fewer Couinaud segments, and major resection as three or more segments [24]. Gas embolism was defined as a ≥ 2 mmHg fluctuation in end-tidal CO₂, based on the diagnostic criteria by Mirski et al. [25]. Postoperative abdominal pain evaluated within 12 h after surgery was categorized into three levels. Level 0: Pain controlled with paracetamol (1360 mg, three times daily) and celecoxib (200 mg, twice daily) alone. Level 1: Pain requiring additional morphine. Level 2: Pain requiring epidural analgesia due to inadequate response to the above drugs.

### Exclusion criteria

Exclusion criteria included general contraindications to insufflation, cardiac or respiratory failure, and/or an American Society of Anesthesiologists physical status of IV. Liver cirrhotic cases with a Child–Pugh score of B and patients with a history of OLS or LLS were excluded.

### RLS surgical technique

The EndoWrist Maryland and fenestrated bipolar forceps were used for minor bleeding control and vessel dissection. Intraoperative ultrasound was routinely employed to identify tumor location and assess the liver remnant. RLS was performed using Trans-parenchymal Glissonian first approach using Robotic Harmonic [26], involving dissection from the caudal or dorsal side to expose the intrahepatic Glissonian pedicle. The console surgeon used a robotic ultrasound drop in probe for real-time identification of target structures. Vessels or bile ducts > 3 mm were clipped with Hem-o-lok, while Glissonian pedicles and hepatic veins > 10 mm were transected using an Endo-GIA stapler via a dorsal approach by the bedside surgeon. Pringle’s maneuver was applied for anatomical resections involving more than one segment. During final haemostasis, PP was reduced to 8 mmHg. The operation concluded after confirming no bleeding or bile leakage from the resection margin.

### Pneumoperitoneal pressure management at Copenhagen University Hospital

In all procedures, the AirSeal® system was utilised to control intra-abdominal pressure (IAP). In the first 130 cases, the standard-PP setting was 12 mmHg. Based on the positive short-term perioperative outcomes experienced at our center [7, 17, 26] and the reported benefits of low-PP [16], IAP of 10 mmHg was adopted as the new standard setting for all RLS. In both groups, any adjustments, such as increasing IAP to control bleeding or stabilize the surgical field, were not performed. During major liver resection requiring prolonged operating time, a standard anaesthetic protocol was followed, with 10-min breaks every 2 h to reduce IAP below 5 mmHg and protect renal and respiratory systems.

### Postoperative care

All patients who underwent liver resection followed the enhanced recovery after surgery program as previously published [27].

### Statistical analysis

Statistical analyses were conducted using IBM SPSS v.26. Fisher’s exact test and Student’s t-test were used for categorical and continuous variables, respectively. Propensity-score matching (1:1 nearest neighbor, no replacement) included age, sex, BMI, ASA class, diagnosis, and extent of resection (minor or major). Continuous variables were reported as mean ± standard deviation or median, depending on distribution. Categorical data were presented as counts (n) or percentages (%). Student’s *t*-test or Mann–Whitney U-test was used for continuous variables, and Chi-squared or Fisher’s exact test for categorical variables. A *p*-value < 0.05 was considered statistically significant.

In this study, the CUSUM analysis was used for the learning curve through JMP Pro 16 (SAS Institute, Cary, NC, USA). The effect of the learning curve on EBL was assessed by CUSUM analysis of consecutive cases in all case, minor resection or major resection. First, patients were ranked consecutively by operative date and the difference from the mean per case was calculated. All data were then weighted and aggregated into difficulty groups, and the CUSUM analysis per case is shown on the y-axis. Learning curves were assessed overall and where significant correlations were found, in a multivariate analysis considering the minor resection group or the major resection group. A graphical representation of the learning curve was depicted to detect the different phases of the learning process (Supplementary Figs. 2a,b,c). In the CUSUM chart, operative cases are presented on the horizontal axis, and the vertical axis represents the standardized cumulative sum of EBL. The upper and lower red lines represent the Upper Control Limit (UCL) and Lower Control Limit (LCL), respectively, defined as follows: UCL: 5, LCL: − 5. Where the unit of UCL or LCL is standard deviation(σ). The limit is set as five times the standard deviation of EBL. The five times the standard deviation uses JMP Pro’s default value.

## Results

### Patient characteristics

Between June 2019 and February 2024, 207 patients underwent RLS (Fig. 1). Fourteen patients were excluded due to lack of data, and 63 cases were assigned to the Low-PP group and 130 cases to the Standard-PP group. After 1:1 PSM, 62 cases remained in each group. Baseline characteristics and outcomes before and after PSM are summarized in Table 1. After PSM, there were no significant differences in age (*p =* 0.619), BMI (26.1 ± 4.5 vs. 25.7 ± 4.6 kg/m^2^; *p =* 0.374), or ASA score. However, Colorectal liver metastases were significantly more common in the Low-PP group (*p <* 0.011). The history of previous abdominal surgery did not differ significantly between the two groups.

**Table 1:** General characteristics of the study groups

	Before PSM			After PSM		
Variable	L-PP (*n* = 63)	S-PP (*n* = 130)	*p*	L-PP (*n* = 62)	S-PP (*n* = 62)	*p*
Age, y, (mean ± SD)	64.5 ± 16.4	63.9 ± 15.7	0.770	64.8 ± 16.3	66.0 ± 15.5	0.619
Gender, male: female	32: 31	68: 62	0.879	32: 30	33: 29	1.000
BMI, kg/m^2^, (mean ± SD)	26.3 ± 4.6	26.1 ± 4.5	0.611	26.1 ± 4.5	25.7 ± 4.6	0.374
ASA score, *n* (%) I II III IV	4/63 (6.3%) 29/63 (46.0%) 29/63 (46.0%) 1/63 (1.6%)	7/130 (5.4%) 63/130 (48.5%) 60/130 (46.2%) 0/130 (0%)	0.751 0.761 1.000 0.326	4/62 (6.5%) 28/62 (45.2%) 29/62 (46.8%) 1/62 (1.6%)	3/62 (4.8%) 29/62 (46.8%) 30/62 (48.4%) 0/62 (0%)	1.000 1.000 1.000 1.000
Diagnosis CRLM HCC/iCC Gallbladder cancer Benign Other	35 (55.6%) 5/3 (12.7%) 7 (11.1%) 6 (9.5%) 6 (9.5%)	43 (33.1%) 26/16 (32.3%) 8 (6.2%) 24 (18.5%) 12 (9.2%)	0.005 0.005 0.257 0.139 1.000	35 (56.5%) 5/3 (12.9%) 7 (11.3%) 6 (9.7%) 6 (9.7%)	20 (32.3%) 15/5 (32.3%) 4 (6.5%) 11 (17.7%) 7 (11.3%)	**0.011** **0.017** 0.530 0.296 1.000
Previous abdominal surgery *n*, (%) Laparotomy *n*, (%) Laparoscopy *n*, (%)	40/63 (63.5%) 10/40 (25.0%) 30/40 (75.0%)	63/130 (48.5%) 13/63 (20.6%) 50/63 (79.4%)	0.065 0.634 0.634	40/62 (64.5%) 10/40 (25.0%) 30/40 (75.0%)	29/62 (46.8%) 9/29 (31.0%) 20/29 (69.0%)	0.070 0.597 0.597

Statistically significant values (*p* < 0.05) are given in bold

Date are expressed as mean ± SD or as number (percentage).

*L-PP* Low pneumoperitoneum pressure, *S-PP* Standard pneumoperitoneum pressure, *BMI*, body mass index, *ASA* American Society of Anesthesiologists, *CRLM* Colorectal liver metastases, *HCC* Hepatocellular carcinoma, *iCC* intrahepatic cholangiocellular carcinoma

### Anesthetic management factors and perioperative outcomes

Perioperative outcomes after PSM are shown in Table 2. Minor and major resection rates, IWATE scores (5.7 ± 2.3 vs. 5.3 ± 2.4; *p =* 0.259) were comparable between groups. PEEP was higher in the Low-PP group (5.0 ± 2.2 vs 3.6 ± 2.5 cm H₂O; *p <* 0.001); no differences were observed in other anesthetic parameters (all *p ≥* 0.05). Pringle maneuver was performed more commonly in the Low-PP group (87.1% vs. 50.0%; *p <* 0.001). In terms of postoperative pain levels, level 0 was significantly more identified in the Standard-PP group (61.3% vs 82.3%; *p =* 0.016). There was no significant difference in EBL (Low-PP group 141.1 ± 162.2 mL vs Standard-PP group 221.5 ± 455.9 mL; *p =* 0.653). Regarding OT, no significant difference was observed between the two groups (Low-PP group 265.3 ± 111.4 min vs Standard-PP group 242.0 ± 110.7 min; *p =* 0.195). In terms of LOS, the two groups also found no significant difference (Low-PP group 3.4 ± 3.3 vs Standard-PP group 3.8 ± 5.2 days; *p =* 0.815). Neither overall complications (Low-PP group 11.3% vs Standard-PP group 12.9%; *p =* 1.000) nor major complications (Low-PP group 6.5% vs Standard-PP group 4.8%; *p =* 1.000) remained significantly different. The incidence of pulmonary complications was comparable in both groups, and neither group experienced episodes of gas embolism. There was no statistically significant difference in the occurrence of acute kidney injury (AKI) between both groups.

**Table 2:** Anaesthesiology parameters and surgical outcomes in all cases

	Before PSM			After PSM		
Variables	L-PP (*n* = 63)	S-PP (*n* = 130)	*p*	L-PP (*n* = 62)	S-PP (*n* = 62)	*p*
Minor resection, *n* (%)	54/63 (85.7%)	98/130 (75.4%)	0.133	54/62 (87.1%)	53/62 (85.5%)	1.000
Major resection, *n* (%)	9/63 (14.3%)	32/130 (24.6%)	0.133	8/62 (12.9%)	9/62 (14.5%)	1.000
IWATE criteria (mean ± SD)	5.7 ± 2.3	5.7 ± 2.4	0.896	5.7 ± 2.3	5.3 ± 2.4	0.259
CVP mmHg, (mean ± SD)	12.2 ± 3.7	13.2 ± 4.6	0.130	12.1 ± 3.7	12.9 ± 4.1	0.217
Et-CO2 mmHg, (mean ± SD)	4.7 ± 0.4	4.7 ± 0.4	0.709	4.7 ± 0.4	4.8 ± 0.4	0.812
PEEP cm H_2_O, (mean ± SD)	5.0 ± 2.2	4.0 ± 2.7	0.008	5.0 ± 2.2	3.6 ± 2.5	**0.001**
Peak airway pressure, cm H_2_O, (mean ± SD)	18.7 ± 2.9	19.8 ± 3.6	0.026	18.7 ± 2.9	19.2 ± 3.6	0.334
Pringle maneuver *n*, (%)	55/63 (87.3%)	66/130 (50.8%)	** < 0.001**	54/62 (87.1%)	31/62 (50.0%)	** < 0.001**
Abdominal pain level *n* (%) 0 1 2	38/63 (60.3%) 16/63 (25.4%) 9/63 (14.3%)	93/130 (71.5%) 26/130 (20.0%) 11/130 (8.5%)	0.140 0.457 0.218	38/62 (61.3%) 15/62 (24.2%) 9/62 (14.5%)	51/62 (82.3%) 7/62 (11.3%) 4/62 (6.5%)	**0.016** 0.098 0.240
Operation time, minutes, median (mean ± SD)	269.3 ± 114.8	265.2 ± 112.3	0.934	265.3 ± 111.4	242.0 ± 110.7	0.195
Estimated blood loss, mL median (mean ± SD)	151.6 ± 181.0	225.5 ± 378.3	0.283	141.1 ± 162.2	221.5 ± 455.9	0.653
Length of stay, days, median (mean ± SD)	3.5 ± 3.3	3.8 ± 4.0	0.424	3.4 ± 3.3	3.8 ± 5.2	0.815
Conversion to open surgery, *n* (%)	0/63 (0%)	3/130 (2.3%)	0.552	0/62 (0%)	2/62 (3.2%)	0.496
Overall complications, *n* (%)	7/63 (11.1%)	27/130 (20.8%)	0.111	7/62 (11.3%)	8/62 (12.9%)	1.000
Minor complication (CD grade 1–2), *n* (%)	3/63 (4.8%)	18/130 (13.8%)	0.082	3/62 (4.8%)	5/62 (8.1%)	0.717
Major complications (CD grade 3–4), *n* (%)	4/63 (6.3%)	9/130 (6.9%)	1.000	4/62 (6.5%)	3/62 (4.8%)	1.000
Subcutaneous emphysema, *n* (%)	0/63 (0%)	3/130 (2.3%)	0.552	0/62 (0%)	1/62 (1.6%)	1.000
Atelectasis/Pneumonia, *n* (%)	0/63 (0%)	2/130 (1.5%)	1.000	0/62 (0%)	1/62 (1.6%)	1.000
Gas embolization, *n* (%)	0/63 (0%)	0/130 (0%)	-	0/62 (0%)	0/62 (0%)	-
Acute kidney injury, *n* (%)	2/63 (3.2%)	7/130 (5.4%)	0.721	2/62 (3.2%)	2/62 (3.2%)	1.000

Statistically significant values (*p* < 0.05) are given in bold

Date are expressed as mean ± SD or as number (percentage)

Abbreviations: *L-PP* Low pneumoperitoneum pressure, *S-PP* Standard pneumoperitoneum pressure, *CD*, Clavien–Dindo classification

### Subgroup analysis of perioperative outcomes in minor liver resection

Perioperative outcomes of minor liver resections are summarized in Supplementary Tables 1. After PSM, IWATE scores were similar between Low-PP and Standard-PP groups (5.2 ± 1.8 vs. 4.7 ± 2.1; *p =* 0.341), and anesthetic parameters were comparable. The rate of Pringle maneuver was statistically significantly more frequent in the Low-PP group (88.9% vs. 53.7%; *p <* 0.001). There were no significant differences between the two groups in terms of EBL, OT, LOS, or complications (all *p ≥* 0.05). The incidence of pulmonary complications or the incidence of acute kidney injury (AKI) was comparable in both groups, and no cases of gas embolism were observed.

### Subgroup analysis of perioperative outcomes in major liver resection

Perioperative outcomes of major liver resections are summarized in Supplementary Tables 2. After PSM, IWATE scores were numerically higher in the Low-PP group, however not significantly different (9.3 ± 1.7 vs. 8.3 ± 1.5; *p =* 0.178). The factors in anesthetic management identified were not different, the Pringle maneuver was performed equally (75.0% vs. 37.5%; *p =* 0.315), and the postoperative pain was comparable. The two groups were comparable regarding EBL, OT, LOS, and complications (all *p ≥* 0.05). No gas embolism, pulmonary complications, or AKI were observed in either group.

### Subgroup analysis of perioperative outcomes in BMI 30 and above

Intraoperative and postoperative outcomes for patients with BMI ≥ 30 are shown in Supplementary Table 3. After PSM, there were no significant differences between the two groups in anesthetic parameters. The Pringle maneuver performed significantly more often in the Low-PP group (93.3% vs. 53.3%; *p =* 0.035). In terms of EBL, OT, LOS, and complications, the two groups were comparable (all *p ≥* 0.05). No cases of pulmonary complications, gas embolism, or acute renal failure were observed in either group.

### Learning curve CUSUM analysis for EBL

Regarding the learning curve CUSUM analysis for EBL, in all cases, initial improvement was apparent around case number 10, followed by a transient upward shift (increased bleeding) observed around cases 70–110, after which levels decreased again. There was no clear, sustained breakpoint. (Supplementary Fig. 2a). As for minor resection, initial improvement was apparent around case number 10. Although fluctuations occurred thereafter, a renewed downward trend was observed around case number 100. (Supplementary Fig. 2b). Following major resection, initial signs of improvement were seen around case number 20. Despite considerable variability thereafter, a renewed decrease was suggested around case number 110 (Supplementary Fig. 2c).

### Discussion

In our PSM cohort study, there were no significant differences in EBL, OT, LOS, or overall/major complications between RLS performed Low- PP (10 mmHg) compared with Standard-PP (12 mmHg). The Pringle maneuver was the more frequently performed in the Low-PP group (87.1% vs 50.0%; *p <* 0.001). There were no cases of gas embolism in each group. These findings suggest that, with advanced robotic systems and stable insufflation technology, the traditional strategy of combining high PP and low CVP may no longer be essential in MILS. Accordingly, it is important to discuss the following three main topics to establish the safety of RLS.

#### PP, CVP, and anesthetic respiratory management in RLS

Based on 54 research papers, the Second International Consensus Conference on Laparoscopic Liver Resection recommends a PP of 12–14 mmHg and low CVP (≤ 5 mmHg) [28]. Conversely, in a pig model, a larger PP–CVP gradient was associated with increased gas embolism risk, with the highest risk typically occurring at PP (12–14 mmHg) + low CVP [29]. In clinical trials, an approach to suppress gas embolism by reducing PP to 8–10 mmHg has also been recommended [30]. Furthermore, several studies have reported an association between high PP and gas embolism occurrence [18, 31–33]. Currently, low CVP management is the standard strategy for OLS [34] and is also recommended for MILS, although disadvantages for ventilation and increased embolism risk have been identified [29–31, 35, 36]. While low airway pressure (e.g., reduced PEEP) may suppress hepatic vein bleeding [41], aligning this with lung-protective ventilation remains a challenge, requiring careful controlled PEEP after recruitment. Furthermore, since the pneumoperitoneum itself can distort CVP values [30], management focused on the “difference” (gradient) between PP and CVP rather than absolute values is considered crucial [19, 29–33, 37–40, 42]. In this study, we did not actively pursue low CVP management at low-PP (10 mmHg), according to the results, maintaining a small PP–CVP gradient. After PSM, including extent of resection (minor or major), EBL showed no difference between 10 and 12 mmHg, and gas embolism was not observed in either group. According to the results, under standardized anesthesia ventilation and hemostasis strategies during MILS, management that avoids excessively widening the PP–CVP gradient may be appropriate for safety and bleeding control. However, prospective trials are necessary to refine the optimal PP/CVP targets for MILS.

#### Inflow control: the impact of the pringle maneuver on EBL under low-PP

The Pringle maneuver is a standard, safe inflow-control technique. At our center it is applied extracorporeally—a 100-cm silk loop passed through a 22-Fr tube via a 12-mm AirSeal® port—allowing rapid occlusion comparable to OLS [7, 17, 26]. Low pneumoperitoneum pressure (PP) can weaken venous tamponade, so mild surface oozing may occur even with standard CVP; in such cases, prophylactic inflow occlusion reduces oozing regardless of PP management. In our study, after PSM analysis including extent of resection (minor or major), EBL did not differ between the Low-PP and Standard-PP groups, whereas Pringle maneuver use was more frequent in the Low-PP group (87.1% vs 50.0%; *p <* 0.001). However, the inflow-control strategy may influence bleeding independently of PP, suggesting residual confounding. Furthermore, timing, total ischemia time, and/or frequency of Pringle maneuver were not standardized, meaning unmeasured aspects of their application may still affect blood loss. In addition, to evaluate potential time-related bias (learning curve/indication expansion), we performed a CUSUM analysis for EBL in all cases, which showed an early improvement (~ case 10), a transient upward deviation during indication expansion (~ cases 70–110), and re-optimization thereafter, without a distinct sustained breakpoint; learning curve on minor or major resection were qualitatively similar (Supplementary Fig. 2a–c). These patterns are consistent with staged programmer adoption and do not suggest a dominant learning curve bias on the primary 10 mmHg vs 12 mmHg PP comparison. However, there may still be unmeasured intraoperative factors (e.g., detailed application of the Pringle maneuver, suction handling, CVP targets). Therefore, prospective studies should identify these hemostatic variables and clarify their interaction with the establishment of PP.

#### Application of robotic instruments in liver parenchyma dissection

Adapting OLS/LLS dissection techniques to RLS remains challenging. This is because CUSA, which cuts and aspirates parenchymal tissue while preserving vessels/lumens, is not compatible with robotic platforms [26, 43]. As both CUSA and Harmonic utilise ultrasonic energy, Robotic Harmonic is adopted as a practical alternative for cutting in RLS in our center. Our technique using Robotic Harmonic for liver parenchymal dissection employs power level 3, with gentle lateral movements of the tip alone under direct field of view, achieving precise parenchymal division with minimal bleeding and reduced risk of venous injury, particularly near major hepatic veins [26]. Hemostasis is enhanced by activating the Harmonic device before complete jaw closure, while superficial oozing is managed using Robotic bipolar coagulation and saline drip administration by the bedside surgeon.

#### Intraoperative techniques to prevent hepatic venous system hemorrhage and gas embolization

Hepatic vein injury is a major risk factor for gas embolism.32,44 Monden et al. classify injuries as “pull-up” (usually controlled with pressure) and “split” at bifurcations/confluences (prone to major bleeding) [44]. We use robotic Harmonic curved shears for gentle root-to-periphery dissection to expose hepatic veins and the Glissonian pedicle while preserving surrounding structures [26]. Stepwise management if bleeding occurs: (1) Pringle maneuver for inflow occlusion. (2) Direct compression of the bleeding point (e.g., Surgicel via a robotic arm). (3) If a major hepatic vein is involved, further parenchymal clearance to fully expose the vein. (4) Clamp with a curved DeBakey bulldog and repair using 4–0/5–0 Prolene. (5) For severe split injuries near the vein origin, early conversion to open surgery should be considered, with PP adjustment and field packing as needed.

### The impact of insufflation devices on low insufflation pressure

Conventional insufflation systems utilize one-way valves for instrument insertion and maintaining pneumoperitoneum pressure. In contrast, AirSeal® achieves stable visualization and IAP even at low pressures and continuous smoke evacuation through its valveless continuous CO₂ flow and three-lumen trocar [45–49]. On the other hand, air entrainment during strong suction and compensatory high-flow CO₂ can be problematic, leading to reports of concerns about gas/air embolism during liver resection. Consequently, some institutions have operational policies restricting or contraindicating the use of AirSeal® on MILS [50–53]. However, the primary determinants of embolization risk are increased PP–CVP gradient, hepatic vein injury, and rapid IAP decrease (and its compensatory overshoot) associated with suction—factors rooted in physiology and operative technique rather than device-specific issues [29, 31–33, 35, 36, 39, 54, 55]. In this study (10–12 mmHg, AirSeal® use), no clinically significant gas embolism was observed, and minimizing the PP–CVP gradient, careful venous handling, and managing suction techniques likely contributed to safety. Consequently, rigorous risk reduction measures based on these principles are essential for any insufflation system. In addition, since this study did not aim for a direct comparison between AirSeal® and conventional devices, we cannot conclude on the superiority of one device over another.

### Post-operative pain related to surgery using low-PP

In this study, abdominal pain level 0 observed more commonly in the standard-PP group than in the Low-PP group (82.3% vs 61.3%, *p =* 0.016). This contrasts with the postoperative pain reduction due to low-PP reported in the review by Ozdemir-van Brunschot et al [16]. Consequently, further research is needed on the specific effects of RLS as a treatment for abdominal pain.

This study has several limitations. First, the between-group difference in pneumoperitoneum was small (2 mmHg), so a type II error remains possible even after matching. Secondly, PSM analysis was performed that included the extent of resection, which reduced residual confounding although it was not eliminated. In particular, hemostatic strategies differed between groups, and key details (Pringle maneuver timing, total ischemia duration, number of Pringle maneuver, suction intensity, CVP targets) were not standardized or captured, which could affect bleeding independent of pressure. Third, a CUSUM analysis for EBL was performed to assess time-related effects; while no distinct, sustained breakpoint was identified, subtle learning effects or practice changes may persist. Fourth, gas/air embolism was monitored clinically (capnography/hemodynamics) without transesophageal echocardiography, so asymptomatic events may have been missed. Fifth, subgroup analyses (e.g., BMI ≥ 30 kg/m^2^) were small and exploratory, and underpowered for definitive comparisons. Sixth, all procedures used AirSeal®, precluding direct comparison with conventional insufflation systems. Finally, the single-center, retrospective design may limit generalizability.

## Conclusion

Our study did not find any statistically significant difference in perioperative outcomes of patients undergoing RLS at a pneumoperitoneal pressure of 10 mmHg versus 12 mmHg. We therefore conclude that performing RLS using a pneumoperitoneal pressure of 10 mmHg PP is feasible and safe. Within standardized RLS programs, PP at 10 mmHg may be a feasible and safe alternative to 12 mmHg PP, although no pressure demonstrated superiority. Determining the optimal target PP requires a multicenter trial with sufficient statistical power.

## Supplementary Information

Below is the link to the electronic supplementary material.

Supplementary file1 (PPTX 119 KB)

Supplementary file2 (PPTX 98 KB)

Supplementary file3 (PPTX 132 KB)

Supplementary file3 (DOCX 41 KB)

## Data Availability

The data that support the findings of this study are available from the corresponding author upon reasonable request.

## Abbreviations

CVP: Central venous pressure
EBL: Estimated blood loss
IAP: Intra-abdominal pressure
Low-PP: Low pneumoperitoneum pressure
LLS: Laparoscopic liver surgery
LOS: Length of hospital stay
MDT: Multidisciplinary team
MILS: Minimally invasive liver surgery
OLS: Open liver surgery
OT: Operative time
PEEP: Positive end-expiratory pressure
PP: Pneumoperitoneum pressure
PSM: Propensity-Score Matched analysis.
RLS: Robotic liver surgery
Standard-PP: Standard pneumoperitoneum pressure
