# Supplementary material for: Evaluating pneumoperitoneum pressure in robotic liver surgery: a propensity-score matched analysis in a high-volume center in Scandinavia
Source: Surg Endosc. 2025 Oct 17;40(1):364–74. doi: 10.1007/s00464-025-12283-2 (PMC12823756; doi:10.1007/s00464-025-12283-2)
Supplement: Supplementary file 1 — Supplementary file1 (PPTX 119 KB) [file 464_2025_12283_MOESM1_ESM.pptx]

## Slide 1
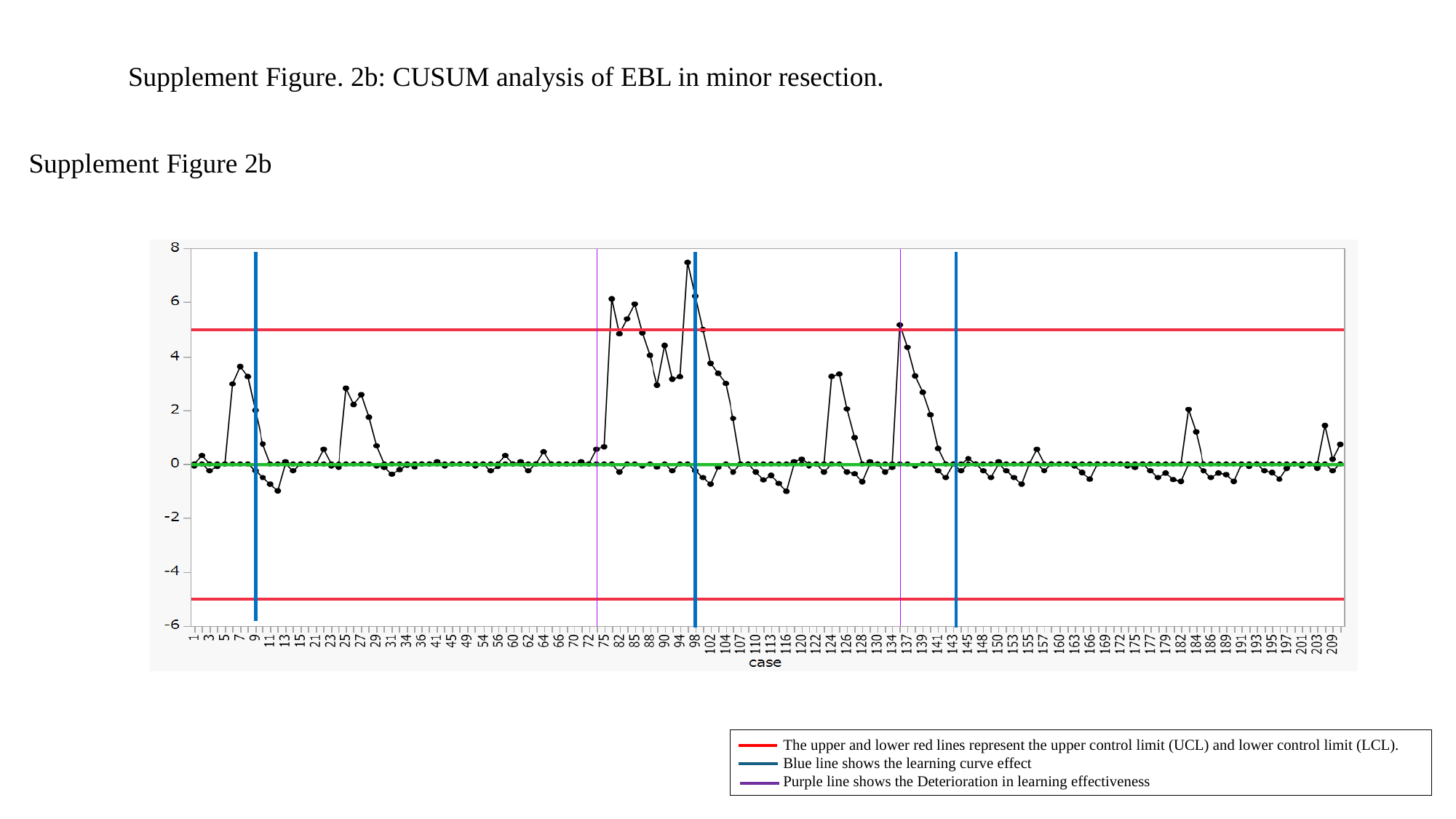

Supplement Figure. 2b: CUSUM analysis of EBL in minor resection.
Supplement Figure 2b
 The upper and lower red lines represent the upper control limit (UCL) and lower control limit (LCL).
 Blue line shows the learning curve effect
 Purple line shows the Deterioration in learning effectiveness
