# Supplementary material for: Evaluating pneumoperitoneum pressure in robotic liver surgery: a propensity-score matched analysis in a high-volume center in Scandinavia
Source: Surg Endosc. 2025 Oct 17;40(1):364–74. doi: 10.1007/s00464-025-12283-2 (PMC12823756; doi:10.1007/s00464-025-12283-2)
Supplement: Supplementary file 3 — Supplementary file3 (PPTX 132 KB) [file 464_2025_12283_MOESM3_ESM.pptx]

## Slide 1
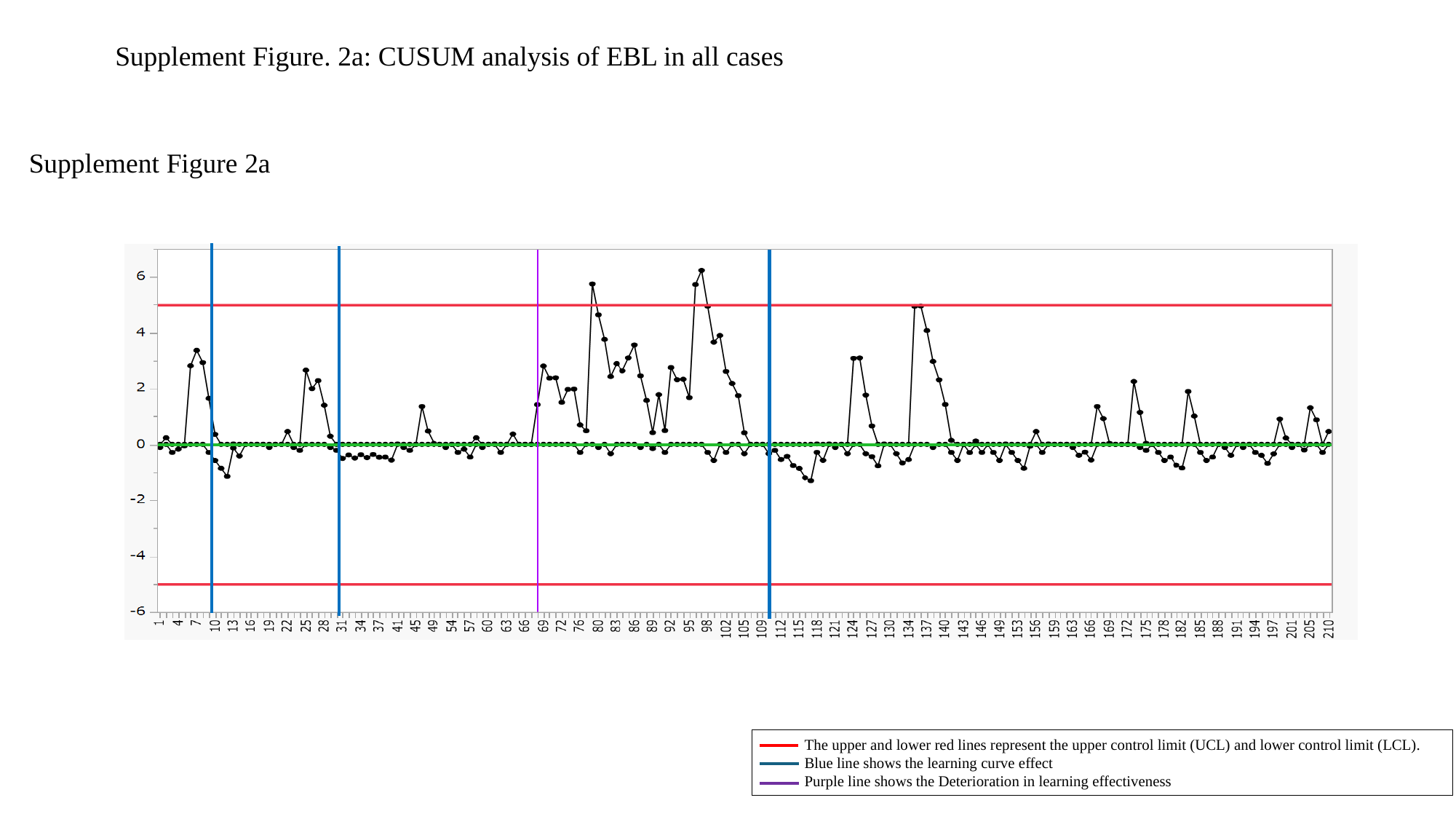

Supplement Figure. 2a: CUSUM analysis of EBL in all cases
Supplement Figure 2a
 The upper and lower red lines represent the upper control limit (UCL) and lower control limit (LCL).
 Blue line shows the learning curve effect
 Purple line shows the Deterioration in learning effectiveness
