# Supplementary material for: Evaluating pneumoperitoneum pressure in robotic liver surgery: a propensity-score matched analysis in a high-volume center in Scandinavia
Source: Surg Endosc. 2025 Oct 17;40(1):364–74. doi: 10.1007/s00464-025-12283-2 (PMC12823756; doi:10.1007/s00464-025-12283-2)
Supplement: Supplementary file 4 — Supplementary file3 (DOCX 41 KB) [file 464_2025_12283_MOESM4_ESM.docx]

Supplementary Tables 1. Anaesthesiology parameters and surgical outcomes in minor resection

|  | Before PSM | |  | After PSM | |  |
| --- | --- | --- | --- | --- | --- | --- |
| Variables | L-PP(n=54) | S-PP (n=98) | p | L-PP (n=54) | S-PP (n=54) | p |
| IWATE criteria (mean ± SD) | 5.2 ± 1.8 | 4.8 ± 2.0 | 0.353 | 5.2 ± 1.8 | 4.7 ± 2.1 | 0.341 |
| CVP mmHg, (mean ± SD) | 12.1 ± 3.7 | 12.9 ± 4.5 | 0.209 | 12.1 ± 3.7 | 12.2 ± 4.6 | 0.711 |
| Et-CO2 mmHg, (mean ± SD) | 4.8 ± 0.5 | 4.8 ± 0.4 | 0.639 | 4.8 ± 0.5 | 4.8 ± 0.4 | 0.980 |
| PEEP cm H_2_O, (mean ± SD) | 5.0 ± 2.2 | 4.2 ± 2.6 | 0.069 | 5.0 ± 2.2 | 4.4 ± 2.4 | 0.156 |
| Peak airway pressure, cm H_2_O (mean ± SD) | 18.6 ± 3.0 | 19.5 ± 3.6 | 0.115 | 18.6 ± 3.0 | 19.3 ± 3.7 | 0.244 |
| Pringle maneuver n, (%) | 48 / 54 (88.9%) | 53 / 98 (54.1%) | **<0.001** | 48 / 54 (88.9%) | 29 / 54 (53.7%) | **<0.001** |
| Abdominal pain level n (%)  0  1  2 | 36 / 54 (66.7%)  12 / 54 (22.2%)  6 / 54 (11.1%) | 74 / 98 (75.5%)  18 / 98 (18.4%)  6 / 98 (6.1%) | 0.260  0.671  0.348 | 36 / 54 (66.7%)  12 / 54 (22.2%)  6 / 54 (11.1%) | 37 / 54 (68.5%)  12 / 54 (22.2%)  5 / 54 (9.3%) | 1.000  1.000  1.000 |
| Operation time, minutes (mean ± SD) | 243.8 ± 92.5 | 233.0 ± 98.7 | 0.444 | 243.8 ± 92.5 | 228.6 ± 99.7 | 0.393 |
| Estimated blood loss, mL (mean ± SD) | 123.0 ± 150.0 | 231.5 ± 420.0 | 0.164 | 123.0 ± 150.0 | 276.1 ± 519.5 | 0.150 |
| Length of stay, days, (mean ± SD) | 2.7 ± 1.5 | 3.3 ± 2.5 | 0.303 | 2.7 ± 1.5 | 3.4 ± 2.6 | 0.314 |
| Conversion to open surgery, n (%) | 0 / 54 (0%) | 3 / 98 (3.1%) | 0.553 | 0 / 54 (0%) | 3 / 54 (5.6%) | 0.243 |
| Overall complications, n (%) | 6 / 54 (11.1%) | 19 / 98 (19.4%) | 0.254 | 6 / 54 (11.1%) | 10 / 54 (18.5%) | 0.417 |
| Minor complication (CD grade 1-2), n (%) | 3 / 54 (5.6%) | 14 / 98 (14.3%) | 0.116 | 3 / 54 (5.6%) | 7 / 54 (13.0%) | 0.320 |
| Major complications (CD grade 3-4), n (%) | 3 / 54 (5.6%) | 5 / 98 (5.1%) | 1.000 | 3 / 54 (5.6%) | 3 / 54 (5.6%) | 1.000 |
| Subcutaneous emphysema, n (%) | 0 / 54 (0%) | 3 / 98 (3.1%) | 0.553 | 0 / 54 (0%) | 2 / 54 (3.7%) | 0.495 |
| Atelectasis / Pneumonia, n (%) | 0 / 54 (0%) | 2 / 98 (2.0%) | 0.539 | 0 / 54 (0%) | 1 / 54 (1.9%) | 1.000 |
| Gas embolization, n (%) | 0 / 54 (0%) | 0 / 98 (0%) | - | 0 / 54 (0%) | 0 / 54 (0%) | - |
| Acute kidney injury, n (%) | 2 / 54 (3.7%) | 2 / 98 (2.0%) | 0.616 | 2 / 54 (3.7%) | 1 / 54 (1.9%) | 1.000 |

Date are expressed as mean ± SD or as number (percentage)

Abbreviations:Low pneumoperitoneum pressure, L-PP; Standard pneumoperitoneum pressure, S-PP; CD, Clavien–Dindo classification

Supplementary Tables 2. Anaesthesiology parameters and surgical outcomes in major resection

|  | Before PSM | |  | After PSM | |  |
| --- | --- | --- | --- | --- | --- | --- |
| Variables | L-PP (n=9) | S-PP (n=32) | p | L-PP (n=8) | S-PP (n=8) | p |
| IWATE criteria (mean ± SD) | 9.2 ± 1.6 | 8.4 ± 1.2 | 0.104 | 9.3 ± 1.7 | 8.3 ± 1.5 | 0.178 |
| CVP mmHg, (mean ± SD) | 12.9 ± 4.1 | 14.1 ± 4.7 | 0.646 | 12.3 ± 3.9 | 12.8 ± 6.3 | 0.790 |
| Et-CO2 mmHg, (mean ± SD) | 4.5 ± 0.3 | 4.6 ± 0.4 | 0.601 | 4.5 ± 0.2 | 4.8 ± 0.4 | 0.185 |
| PEEP cm H_2_O, (mean ± SD) | 5.2 ± 2.2 | 3.4 ± 2.9 | **0.043** | 5.0 ± 2.2 | 1.9 ± 2.6 | **0.021** |
| Peak airway pressure, cm H_2_O, (mean ± SD) | 19.2 ± 2.2 | 20.8 ± 3.1 | 0.221 | 19.4 ± 2.3 | 20.0 ± 2.6 | 0.789 |
| Pringle maneuver, n (%) | 7 / 9 (77.8%) | 13 / 32 (40.6%) | **0.067** | 6 / 8 (75.0%) | 3 / 8 (37.5%) | 0.315 |
| Abdominal pain level n (%)  0  1  2 | 2 / 9 (22.2%)  4 / 9 (44.4%)  3 / 9 (33.3%) | 19 / 32 (59.4%)  8 / 32 (25.0%)  5 / 32 (15.6%) | 0.067  0.408  0.342 | 2 / 8 (25.0%)  3 / 8 (37.5%)  3 / 8 (37.5%) | 5 / 8 (62.5%)  1 / 8 (12.5%)  2 / 8 (25.0%) | 0.315  0.569  1.000 |
| Operation time, minutes, (mean ± SD) | 441.4 ± 106.2 | 365.8 ± 91.5 | 0.095 | 431.1 ± 110.4 | 388.3 ± 73.5 | 0.487 |
| Estimated blood loss, mL (mean ± SD) | 323.3 ± 257.2 | 207.2 ± 210.0 | 0.193 | 263.8 ± 197.7 | 145.6 ± 202.2 | 0.167 |
| Length of stay, days, (mean ± SD) | 7.9 ± 6.4 | 5.3 ± 6.7 | **0.015** | 8.3 ± 6.7 | 4.0 ± 1.7 | 0.054 |
| Conversion to open surgery, n (%) | 0 / 9 (0%) | 0 / 32 (0%) | - | 0 / 8 (0%) | 0 / 8 (0%) | - |
| Overall complications | 1 / 9 (11.1%) | 8 / 32 (25.0%) | 0.654 | 1 / 8 (12.5%) | 2 / 8 (25.0%) | 1.000 |
| Minor complication (CD grade 1-2), n (%) | 0 / 9 (0%) | 4 / 32 (12.5%) | 0.559 | 0 / 8 (0%) | 1 / 8 (12.5%) | 1.000 |
| Major complications (CD grade 3-4), n (%) | 1 / 9 (11.1%) | 4 / 32 (12.5%) | 1.000 | 1 / 8 (12.5%) | 1 / 8 (12.5%) | 1.000 |
| Subcutaneous emphysema, n (%) | 0 / 9 (0%) | 0 / 32 (0%) | - | 0 / 8 (0%) | 0 / 8 (0%) | - |
| Atelectasis / Pneumonia, n (%) | 0 / 9 (0%) | 0 / 32 (0%) | - | 0 / 8 (0%) | 0 / 8 (0%) | - |
| Gas embolization, n (%) | 0 / 9 (0%) | 0 / 32 (0%) |  | 0 / 8 (0%) | 0 / 8 (0%) | - |
| Acute kidney injury, n (%) | 0 / 9 (0%) | 5 / 32 (15.6%) | 0.568 | 0 / 8 (0%) | 0 / 8 (0%) |  |

Date are expressed as mean ± SD or as number (percentage)

Abbreviations:Low pneumoperitoneum pressure, L-PP; Standard pneumoperitoneum pressure, S-PP; CD, Clavien–Dindo classification

Supplementary Tables 3. Analysis in the subgroup of obese with BMI greater than 30 kg/m^2^

|  | Before PSM | |  | After PSM | |  |
| --- | --- | --- | --- | --- | --- | --- |
| Variables | L-PP (n=16) | S-PP (n=23) | p | L-PP (n=15) | S-PP (n=15) | p |
| Age, y (mean ± SD) | 61.9 ± 20.3 | 63.4 ± 12.6 | 0.819 | 63.0 ± 20.5 | 62.5 ± 14.6 | 0.633 |
| Previous surgery yes, n (%) | 6 / 16 (37.5%) | 10 / 23 (43.5%) | 0.752 | 6 / 15 (40.0%) | 4 / 15 (26.7%) | 0.700 |
| ASA score,n (%)  I  II  III  IV | 0 / 16 (0%)  7 / 16 (43.8%)  9 / 16 (56.3%)  0 / 16 (0%) | 1 / 23 (4.3%)  10 / 23 (43.5%)  12 / 23 (52.2%)  0 / 23 (0%) | 1.000  1.000  1.000  - | 0 / 15 (0%)  6 / 15 (40.0%)  9 / 15 (60.0%)  0 / 15 (0%) | 1 / 15 (6.7%)  7 / 15 (46.7%)  7 / 15 (46.7%)  0 / 15 (0%) | 1.000  1.000  0.715  - |
| CVP mmHg, (mean ± SD) | 12.6 ± 4.1 | 14.4 ± 5.0 | 0.252 | 12.3 ± 4.0 | 13.8 ± 4.7 | 0.317 |
| Et-CO2 kPa mmHg, (mean ± SD) | 4.8 ± 0.5 | 4.8 ± 0.4 | 0.595 | 4.8 ± 0.5 | 4.7 ± 0.3 | 0.786 |
| PEEP cm H_2_O, (mean ± SD) | 4.9 ± 2.6 | 4.5 ± 3.4 | 0.610 | 4.7 ± 2.6 | 5.5 ± 2.7 | 0.552 |
| Peak airway pressure, cm H_2_O, (mean ± SD) | 20.6 ± 2.3 | 22.4 ± 3.3 | 0.137 | 20.8 ± 2.2 | 22.3 ± 2.9 | 0.153 |
| Pringle maneuver, n (%) | 15 / 16 (93.8%) | 10 / 23 (43.5%) | **0.002** | 14 / 15 (93.3%) | 8 / 15 (53.3%) | **0.035** |
| Abdominal pain level, n (%)  0  1  2 | 11 / 16 (68.8%)  3 / 16 (18.8%)  2 / 16 (12.5%) | 16 / 23 (69.6%)  5 / 23 (21.7%)  2 / 23 (8.7%) | 1.000  1.000  1.000 | 11 /15 (73.3%)  2 / 15 (13.3%)  2 / 15 (13.3%) | 10 / 15 (66.7%)  4 / 15 (26.7%)  1 / 15 (6.7%) | 1.000  0.651  1.000 |
| Operation time, minutes, (mean ± SD) | 313.9 ± 126.0 | 280.7 ± 113.8 | 0.424 | 300.7 ± 118.3 | 260.1 ± 111.7 | 0.330 |
| Estimated blood loss, mL (mean ± SD) | 240.0 ± 250.1 | 228.0 ± 241.1 | 0.954 | 202.7 ± 207.6 | 227.7 ± 243.6 | 0.834 |
| Length of stay, days (mean ± SD) | 3.2 ± 1.7 | 3.5 ± 3.2 | 0.701 | 3.1 ± 1.7 | 3.0 ± 2.6 | 0.480 |
| Conversion to open surgery, n (%) | 0 / 16 (0%) | 1 / 23 (4.3%) | 1.000 | 0 / 15 (0%) | 1 / 15 (6.7%) | 1.000 |
| Overall complications n (%) | 0 / 16 (0%) | 6 / 23 (26.1%) | 0.064 | 0 / 15 (0%) | 3 / 15 (20.0%) | 0.224 |
| Minor complication (CD grade 1-2), n (%) | 0 / 16 (0%) | 2 / 23 (8.7%) | 0.503 | 0 / 15 (0%) | 1 / 15 (6.7%) | 1.000 |
| Major complications (CD grade 3-4), n (%) | 0 / 16 (0%) | 4 / 23 (17.4%) | 0.130 | 0 / 15 (0%) | 2 / 15 (13.3%) | 0.483 |
| Subcutaneous emphysema, n (%) | 0 / 16 (0%) | 0 / 23 (0%) | - | 0 / 15 (0%) | 0 / 15 (0%) | - |
| Atelectasis / Pneumonia, n (%) | 0 / 16 (0%) | 0 / 23 (0%) | - | 0 / 15 (0%) | 0 / 15 (0%) | - |
| Gas embolization, n (%) | 0 / 16 (0%) | 0 / 23 (0%) | - | 0 / 15 (0%) | 0 / 15 (0%) | - |
| Acute kidney injury, n (%) | 0 / 16 (0%) | 2 / 23 (8.7%) | 0.503 | 0 / 15 (0%) | 1 / 15 (6.7%) | 1.000 |

Date are expressed as mean ± SD or as number (percentage)

Abbreviations:Low pneumoperitoneum pressure, L-PP; Standard pneumoperitoneum pressure, S-PP; CD, Clavien–Dindo classification
